# Impact of COVID-19 on Pregnancy Outcomes: A Phase-Based Analysis from a Spanish Tertiary Hospital (2020–2023)

**DOI:** 10.3390/jcm14145136

**Published:** 2025-07-19

**Authors:** María-Asunción Quijada-Cazorla, María-Virgilia Simó-Rodríguez, Ana-María Palacios-Marqués, María Peláez-García, José-Manuel Ramos-Rincón

**Affiliations:** 1 Department of Obstetrics and Gynecology, Dr. Balmis General University Hospital, 03010 Alicante, Spain; apalacios@umh.es (A.-M.P.-M.);; 2 Alicante Institute for Health and Biomedical Research (ISABIAL), 03010 Alicante, Spain; jose.ramosr@umh.es; 3 Department of Public Health, History of Science and Gynecology, Miguel Hernández University, 03550 San Juan de Alicante, Spain; 4 Department of Internal Medicine, Dr. Balmis General University Hospital, 03010 Alicante, Spain; 5 Department of Clinical Medicine, Miguel Hernández University, 03550 San Juan de Alicante, Spain

**Keywords:** COVID-19, SARS-CoV-2, pregnancy, cesarean delivery, maternal outcomes, neonatal outcomes, risk factors, vaccination

## Abstract

**Background/Objectives:** Pregnancy has been considered a risk factor for severe SARS-CoV-2 infection, as well as for adverse maternal and neonatal outcomes. This study aimed to assess the clinical impact of COVID-19 on pregnant women managed at a Spanish tertiary care hospital across different phases of the pandemic. **Methods:** A retrospective observational study was conducted at Dr. Balmis General University Hospital (Alicante, Spain) between March 2020 and May 2023. All pregnant women who received hospital care with confirmed SARS-CoV-2 infection were included. Maternal and neonatal outcomes were analyzed and compared with the 6120 total births recorded during the same period. **Results:** A total of 249 pregnant women with COVID-19 were included, with 30.8%, 25.0%, and 7.9% hospitalized during each respective pandemic phase. The overall incidence of infection was 41 cases per 1000 births. Hospitalized pregnant women showed significantly higher rates of preterm birth, labor induction (70.4% vs. 47.0%; OR: 2.67; 95% CI: 1.12–6.43), and cesarean delivery (46.9% vs. 24.9%, OR: 2.60; 95% CI: 1.27–5.50). Neonatal outcomes included lower Apgar scores, increased admission to the neonatal unit (25.8% vs. 8.2%, *p* = 0.007), and a higher rate of neonatal complications (23.3% vs. 7.7%, *p* = 0.015). Maternal obesity and non-Spanish nationality were associated with more severe maternal disease. Vaccination against SARS-CoV-2 significantly reduced the risk of hospitalization due to the infection (OR: 0.30; 95% CI: 0.13–0.69). **Conclusions:** Pregnant women admitted with COVID-19 had increased risks of adverse obstetric and neonatal outcomes, underscoring the importance of preventive strategies, such as vaccination.

## 1. Introduction

Since the World Health Organization (WHO) declared COVID-19 a global pandemic on 11 March 2020, its impact on pregnancy has become a significant clinical concern due to its potential maternal and perinatal consequences. Pregnancy has been recognized as a risk factor for more severe SARS-CoV-2 infection as well as for adverse obstetric and neonatal outcomes [1]. Although most pregnant women presented with mild symptoms, with 73–86% remaining asymptomatic [2], approximately 16% progressed to severe disease [3].

Throughout the pandemic, the literature has reported variable effects of this coronavirus on maternal and neonatal health. Multiple studies have shown that SARS-CoV-2 infection during pregnancy is associated with higher maternal mortality [4], an increased risk of intensive care unit (ICU) admission compared to nonpregnant women, and up to a 3.5-fold increase in hospitalization rates [5,6,7]. Advanced maternal age, elevated body mass index (BMI), non-White ethnicity, and preexisting comorbidities have been associated with a worse prognosis [5].

The impact of SARS-CoV-2 on pregnancy-related outcomes has also been extensively discussed. Several studies have reported increased cesarean delivery rates [6,8] and higher risks of pregnancy-associated complications, including gestational diabetes, hypertensive disorders, and preterm labor [8,9]. In contrast, no significant differences have been observed in the incidence of other obstetric complications, such as postpartum hemorrhage [10].

Importantly, across the different phases of the pandemic, each SARS-CoV-2 variant has been associated with distinct clinical implications. The original Wuhan wild-type strain was initially considered the most virulent, with potentially more severe clinical outcomes involving the highest rate of cesarean deliveries [11]. The Delta variant demonstrated approximately 60% greater transmissibility than its predecessor and was linked to a significantly higher incidence of adverse clinical events [11,12]. In contrast, the Omicron variant, although more transmissible, was generally associated with milder clinical manifestations compared to earlier variants [11,13]. Despite these findings, few studies have explored the evolving impact of COVID-19 across the different pandemic phases, variant waves, and their specific implications for pregnancy.

In summary, the COVID-19 pandemic has presented a significant challenge to maternal and perinatal health, with an impact that remains incompletely characterized. Although the acute phase of the pandemic has passed, limited evidence is available regarding the progression of its clinical effects across successive viral variants, the role of vaccination, and its overall consequences for pregnancy. To address these gaps, we conducted a study to evaluate the clinical outcomes of SARS-CoV-2 infection during pregnancy, as well as its evolution throughout the different phases of the pandemic.

This study aims to provide real-world evidence of the temporal progression of the COVID-19 pandemic and its impact on maternal and perinatal outcomes at a Spanish tertiary care hospital from 2020 to 2023.

## 2. Materials and Methods

### 2.1. Study Design and Type

A retrospective cohort study was conducted, including all pregnant women diagnosed with COVID-19 during pregnancy who received care for this reason at Dr. Balmis General University Hospital (DBGUH) in Alicante, Spain, between 11 March 2020 and 5 May 2023. DBGUH is a tertiary-level referral center equipped with a neonatal intensive care unit (NICU) and serving a healthcare area of approximately 290,000 inhabitants. In addition to providing routine obstetric care, the hospital manages high-risk pregnancies, particularly those in which neonatal complications are anticipated and NICU admission is expected.

### 2.2. Study Population

Inclusion criteria: Those considered eligible for participation in this study fit the following criteria: pregnant women who received care at DBGUH and were diagnosed with SARS-CoV-2 infection using reverse transcription polymerase chain reaction (RT-PCR) during pregnancy between 11 March 2020 and 5 May 2023, and who subsequently gave birth at the same hospital. Therefore, moderate-to-severe cases of pregnant women with confirmed SARS-CoV-2 infection and COVID-19-related symptoms who attended the hospital emergency department for this reason were included.

All COVID-19 diagnoses included in the study were confirmed through reverse transcription polymerase chain reaction (RT-PCR) performed by medical professionals at the hospital. Home-based self-tests were not included, as the study was based on hospital medical records and laboratory-confirmed cases.

Exclusion criteria: The following cases were excluded: (1) patients incorrectly coded with COVID-19 who were not pregnant at the time of infection, and (2) pregnant women in whom SARS-CoV-2 infection was ruled out or could not be microbiologically confirmed.

The study group was compared in terms of obstetric outcomes with a control group. The inclusion criteria for the control group consisted of pregnant women without a confirmed SARS-CoV-2 infection (either a negative COVID-19 PCR test or no available test results) who gave birth at the same tertiary hospital during the study period, and who were not treated in the emergency department for COVID-19 infection during pregnancy.

### 2.3. Study Periods and Phase Definitions

Three study periods were defined based on the predominant circulating SARS-CoV-2 variants and the progression of the national COVID-19 vaccination campaign in Spain, according to epidemiological surveillance data:

- Period 1 (11 March 2020–28 February 2021): Characterized by the original Wuhan strain and a non-vaccinated population. 28 February 2021 was selected as the cutoff point because it marked the beginning of sustained circulation of the Alpha variant in Spain and the initial rollout of COVID-19 vaccination in pregnant women.
- Period 2 (1 March 2021–31 December 2021): Defined by the predominance of the Delta variant and a partially vaccinated population. During this phase, vaccination coverage among pregnant women began to progressively increase.
- Period 3 (1 January 2022–5 May 2023): Corresponding to the predominance of the Omicron variant and widespread vaccination coverage. This period was associated with high vaccination rates and reduced COVID-19 severity, in line with the evolving behavior of circulating variants and the national public health response.

These cutoff dates were selected to reflect key epidemiological and clinical transitions throughout the pandemic that may have influenced maternal and neonatal outcomes.

The results for each study period were compared with those recorded for a cohort of pregnant women without COVID-19 who delivered at the same hospital during the corresponding timeframe.

### 2.4. Data Collection and Definitions

Clinical data were obtained from electronic medical records and systematically recorded in an anonymized data collection form.

The following variables were collected:

- Maternal history: Maternal age, gestational age at the time of infection, country of birth, height, weight, body mass index (BMI), pregestational comorbidities (e.g., chronic respiratory disease, chronic hypertension, pregestational diabetes), COVID-19 vaccination status, parity, and history of previous spontaneous abortion.
- COVID-19-related variables: Symptoms (fever, cough, sore throat, dyspnea, myalgia, anosmia), emergency department visits, hospitalization, length of hospital stay, ICU admission and length of stay, treatments received (low molecular weight heparin, corticosteroids, oxygen therapy), and complications (bilateral pneumonia, thromboembolic events, maternal death, use of invasive or non-invasive mechanical ventilation).
- Pregnancy-related variables: Pregnancy outcome (miscarriage, preterm delivery, or term delivery), gestational complications (gestational diabetes, hypertensive disorders, threatened preterm labor), and fetal conditions (intrauterine growth restriction (IUGR)/small for gestational age (SGA), congenital anomalies, or other fetal complications).
- Delivery and perinatal outcomes: Date of delivery, gestational age at delivery, onset of labor (spontaneous or induced), and mode of delivery (vaginal or cesarean).
- Maternal postpartum complications: Postpartum hemorrhage, need for maternal blood transfusion, postpartum fever, ICU admission, and other maternal complications.
- Neonatal outcomes: Birth weight, Apgar scores, arterial and venous umbilical cord pH, NICU admission, perinatal complications, and neonatal death.

The two primary outcomes of the study focused on pregnant women with confirmed SARS-CoV-2 infection. The first outcome was the need for hospital admission after emergency department evaluation. The second outcome was the development of severe maternal COVID-19, defined as ICU admission, the use of invasive or non-invasive mechanical ventilation (IMV/NIMV), and/or maternal death.

### 2.5. Statistical Analysis

Statistical analyses were performed using IBM SPSS Statistics for Windows, version 25.0 (IBM Corp., Armonk, NY, USA). All variables were subjected to descriptive analysis. The distribution of quantitative variables was assessed using the Kolmogorov–Smirnov test. Variables with a normal distribution were reported as mean and standard deviation (SD), while non-normally distributed variables were expressed as the median and interquartile range (IQR). Categorical variables were summarized as absolute frequencies and percentages.

To compare quantitative variables between groups, Student’s *t*-test was used for normally distributed variables and the Mann–Whitney U test was applied for non-normally distributed ones. For categorical variables, comparisons were performed using the chi-square test or Fisher’s exact test, as appropriate. The strength of association was quantified using odds ratios (ORs) with 95% confidence intervals (95% CI). A *p*-value < 0.05 was considered statistically significant.

A complete case analysis was conducted for each variable. Due to the retrospective nature of the study and the variability in the completeness of clinical records, the number of available observations (N) differed slightly between variables.

### 2.6. Ethical Approach

The study was conducted following the ethical principles of the Declaration of Helsinki. The protocol was approved by the Ethics Committee of the Alicante Health Department-General Hospital, with project identification code CEIm PI2024-036/ISABIAL 2024-0049, on 7 March 2024. All data were collected in an anonymized form, and patient confidentiality was rigorously maintained throughout the study.

## 3. Results

### 3.1. COVID-19 in Pregnant Women

A total of 319 pregnant women diagnosed with SARS-CoV-2 infection during pregnancy and evaluated in the emergency department were initially identified.

**Figure 1:**
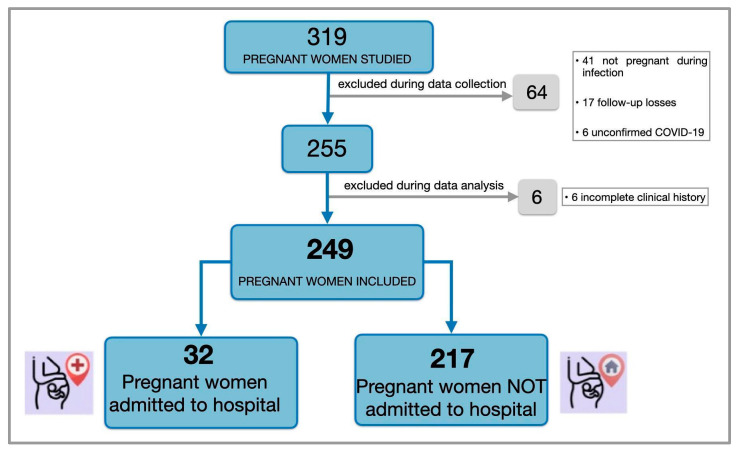
Flowchart of the inclusion and exclusion process for pregnant women with confirmed. SARS-CoV-2 infection during the study period. This figure represents the selection of the COVID-19 cohort only.

After applying the exclusion criteria, 70 women were excluded, resulting in a final study population of 249 pregnant women for analysis. The included and excluded patients are presented in Figure 1. Figure 1 illustrates the selection process of the study cohort: A total of 319 pregnant women were initially identified with a diagnosis of COVID-19 during pregnancy. Of these, 70 were excluded due to not meeting the inclusion criteria (e.g., incomplete data, delivery outside the hospital), and 9 experienced a miscarriage before 22 weeks of gestation. Therefore, the final study cohort—meeting all inclusion and exclusion criteria—consisted of 240 pregnant women with confirmed SARS-CoV-2 infection whose pregnancies progressed beyond 22 weeks and who delivered at the hospital.

**Figure 2:**
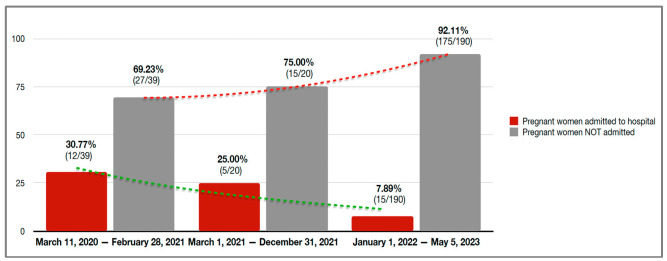
Number and percentage of pregnant women with confirmed COVID-19 and hospital admission rates across the different phases of the pandemic.

Between 11 March 2020, and 28 February 2021 (Period 1), 39 women (15.7%) were managed. Between March 1 2021 and 31 December 2021 (Period 2), 20 women (8.0%) were included. Most cases occurred between 1 January 2022 and 5 May 2023 (Period 3), involving 190 women (76.3%). Overall, 12.9% of the pregnant women evaluated in the emergency department required hospitalization. The hospital admission rates were 30.8% in Period 1, 25.0% in Period 2, and 7.9% in Period 3. These trends are shown in Figure 2.

### 3.2. Obstetric Outcomes: COVID-19 vs. Non-COVID-19 Pregnancies

During the study period, a total of 6120 deliveries occurred at the hospital. Of these, 5880 involved pregnant women without COVID-19, whose deliveries took place at the hospital but who did not meet the inclusion criteria for the study cohort. The remaining 240 deliveries involved pregnant women with confirmed SARS-CoV-2 infection during pregnancy, all of whom were evaluated and managed at the hospital’s obstetric emergency department, and whose deliveries also occurred at the same institution. This corresponds to an incidence of 4.1%, or 41 cases per 1000 deliveries.

Of the initial 249 COVID-19 cases managed at the hospital (Figure 1), 9 pregnancies ended in miscarriage before 22 weeks of gestation and were excluded from the analysis. The final study cohort included 240 pregnant women who met all the eligibility criteria.

Pregnant women with COVID-19 showed a significantly higher rate of preterm birth compared to the non-COVID-19 group (18.3% vs. 8.7%, *p* < 0.001). Comparative results between both groups, stratified by gestational age and study period, are presented in Table 1.

Cesarean section rates were higher in COVID-19 pregnancies across all periods, although the differences were not statistically significant: 36.1% vs. 23.3% in Period 1 (*p* = 0.072); 33.3% vs. 23.8% in Period 2 (*p* = 0.181); and 27.4% vs. 24.1% in Period 3 (*p* = 0.321). The overall cesarean section rate approached statistical significance: 29.2% vs. 23.8% (*p* = 0.052).

The induction of labor was also more frequent among patients with COVID-19 across all periods: 47.2% vs. 40.3% in Period 1 (*p* = 0.411), 66.7% vs. 41.1% in Period 2 (*p* = 0.028), and 41.5% vs. 34.9% in Period 3 (*p* = 0.075). Table 1 presents detailed data on cesarean sections and labor inductions for each phase.

### 3.3. Epidemiological and Clinical Characteristics in Pregnant Women with COVID-19: Admitted vs. Non-Admitted

Of the 249 pregnant women included, 32 (12.9%) required hospitalization due to COVID-19. Table 2 summarizes the epidemiological characteristics, comorbidities, clinical symptoms, complications, and outcomes among the pregnant women with COVID-19 evaluated in the emergency department, comparing those who were admitted to those who were not. The proportion of admissions was higher during the first and second periods of the study (37.0% and 15.6% vs. 12.4% and 6.9%), whereas it was lower in the third period (46.9% vs. 80.6%; *p* < 0.001). A history of pulmonary disease was significantly more frequent among admitted pregnant women (15.6% vs. 5.1%, *p* = 0.04).

COVID-19-related symptoms (such as fever, cough, myalgia, odynophagia, dyspnea, and anosmia) were more commonly reported in hospitalized women. Notably, pneumonia was diagnosed in 25% of admitted patients, whereas none of the non-admitted patients developed pneumonia (*p* < 0.001).

### 3.4. Obstetric Outcomes in Pregnant Women with COVID-19: Admitted vs. Non-Admitted

Obstetric outcomes according to hospitalization status are detailed in Table 3. Hospitalized pregnant women were more frequently at earlier gestational ages (<24 and 24–34 weeks) and less commonly at ≥ 35 weeks compared to non-hospitalized women (*p* = 0.037).

The obstetric history and pregnancy-related complications were similar in both groups. However, the rates of induced labor (59.4% vs. 39.6%, *p* = 0.023) and cesarean section (46.9% vs. 24.9%, *p* = 0.009) were significantly higher in hospitalized patients. Maternal admission to the intensive care unit (ICU) due to pregnancy-related complications from COVID-19 occurred exclusively in admitted women (9.7% vs. 0%, *p* < 0.002). Full results are provided in Table 3.

### 3.5. Neonatal Outcomes in Pregnant Women with COVID-19: Admitted vs. Non-Admitted

Birth weight did not differ significantly between groups. However, neonates born to hospitalized mothers had significantly lower Apgar scores at 1 min (18.7% vs. 2.9%, *p* < 0.001), higher NICU admission (25.8% vs. 8.2%, *p* = 0.007), and more neonatal complications (23.3% vs. 7.7%, *p* = 0.015). Detailed findings are shown in Table 4.

### 3.6. Risk Factors for Severe COVID-19 Among Pregnant Women Admitted

Of the 32 pregnant women who were hospitalized, clinical data were unavailable for one patient. Therefore, the analysis included 31 women, of whom 5 (16.1%) experienced a severe episode, defined as ICU admission (n = 3), requirement for invasive or non-invasive mechanical ventilation (IMV/NIMV) (n = 5), and/or death (n = 1). The mean length of hospital stay for these patients was 15.4 days (SD ± 22.6).

Risk factors significantly associated with severe COVID-19 included non-Spanish nationality (100% vs. 38.5%, *p* = 0.018) and obesity (60% vs. 12%, *p* = 0.041). Detailed data are presented in Table 5.

### 3.7. Effect of Vaccination on Hospital Admission in Pregnant Women

Among the hospitalized pregnant women, 71.9% were unvaccinated, compared to 56.2% of those not admitted who had received at least one dose of a COVID-19 vaccine. This difference was statistically significant (OR = 0.30; 95% CI: 0.13–0.69), indicating a protective effect of vaccination against hospital admission. Detailed results are provided in Table 6.

## 4. Discussion

This study provides important insights into the clinical characteristics, obstetric outcomes, and neonatal complications associated with COVID-19 among pregnant women in Spain, a country that experienced a particularly high burden of infections and hospitalizations during the pandemic. Although the overall incidence of COVID-19 in our cohort remained relatively low, it increased markedly during the Omicron wave, likely due to the relaxation of public health measures and the higher transmissibility of this variant. Severe maternal infection was associated with complications such as preeclampsia, preterm birth, and cesarean delivery, emphasizing the importance of close maternal monitoring. Neonatal outcomes—particularly NICU admissions—were more frequent among neonates born to women with severe disease, consistent with previous studies [6]. Additionally, COVID-19 vaccination emerged as a protective factor against severe disease and hospitalization, underscoring the importance of immunization during pregnancy.

### 4.1. Prevalence and Incidence of COVID-19

The incidence of COVID-19 among pregnant women during the study period (11 March 2020–5 May 2023), based on total deliveries, was 4.1%. However, this rate is lower than that reported in other studies. For instance, a meta-analysis [5] found an incidence of up to 10% among pregnant women who presented to or were admitted to hospitals during the early stages of the pandemic. Similarly, a large retrospective cohort study by Son et al. [13] reported an SARS-CoV-2 positivity rate of 6.9% in pregnant women between March and December 2020.

The lower incidence observed in our study may be explained by our inclusion criteria, which focused on pregnant women who received hospital care for COVID-19-related symptoms—representing more severe cases—rather than on all pregnant women with COVID-19. This likely excluded many asymptomatic or mildly symptomatic cases managed in outpatient settings.

By pandemic phase, incidence rates were 20.6 and 12.3 per 1000 deliveries during the first and second phases, respectively, closely aligning with Donati et al. [14], who reported rates of 23.5 and 16.6 per 1000 deliveries during the same periods. However, a substantial increase was observed during the third phase, with the incidence rate rising to 73.2 per 1000 deliveries. These findings are consistent with data from England, where a population-based study by Vousden et al. [15] confirmed increases in infection and disease severity during the Delta and Omicron waves compared to the wild-type and Alpha variants. This surge is likely attributable to the combination of relaxed public health measures and the greater transmissibility of the Omicron variant compared to previous strains.

In relation to SARS-CoV-2 variants, studies such as the Italian study by Incognito et al. [11] found that the Delta variant was linked to the most severe maternal and neonatal outcomes, including higher rates of ICU admission, preterm birth, and mortality. Alpha showed intermediate severity, while Omicron was associated with milder disease.

### 4.2. Obstetric Outcomes: COVID-19 vs. Non-COVID-19 Pregnancies

Over the 3 years and 2 months of the COVID-19 pandemic covered by this study, we observed an increased incidence of preterm birth among pregnant women with SARS-CoV-2 infection. Although this rate declined across pandemic waves, it remained elevated during the third wave (13.44%), compared to the 8.43% observed among non-infected pregnant women. These results align with prior studies reporting an increased risk of preterm delivery associated with SARS-CoV-2 infection, particularly during the initial phases of the pandemic [9,14,16]. Our findings are also consistent with Seaton et al. [17], who reported a reduced preterm birth rate during the Omicron period compared to earlier waves. This reduction may be attributed to the lower virulence of the Omicron variant and the protective effect of COVID-19 vaccination, as highlighted by Hui et al. [18].

The relationship between COVID-19 and the mode of delivery has evolved over time. Early in the pandemic, some studies reported an increased rate of cesarean sections [11,17,19]. However, more recent evidence has not supported a significant association between SARS-CoV-2 infection and delivery mode [6,14,20]. The results of our study are consistent with the latter body of evidence, as we did not observe a statistically significant increase in cesarean deliveries among infected pregnant women.

The impact of SARS-CoV-2 infection on labor induction has been less extensively studied. In our cohort, a rising trend in induction rates was observed among infected pregnant women, reaching statistical significance exclusively during the second wave of the pandemic. Other authors have similarly reported increased rates of labor induction and planned cesarean sections among pregnant women with moderate-to-severe COVID-19 [21,22]. This trend probably indicates the need for closer monitoring and proactive obstetric management in infected patients.

### 4.3. Epidemiological Characteristics and Clinical Outcomes: Admitted vs. Non-Admitted Pregnant Women

In our cohort, 12.9% of pregnant women diagnosed with COVID-19 required hospitalization due to the infection. A significant variation in hospitalization rates was observed across different phases of the pandemic, with more admissions during the first and third waves.

A single case of maternal death was reported, corresponding to a maternal mortality rate of 0.4%, which is comparable to the 0.1% reported by Jering et al. [23]. However, the overall in-hospital mortality rate among pregnant women in our cohort was 3.1%, notably higher than the 1.1% reported by Pineles et al. [7]. This discrepancy may be attributable to the timing of infection, as most severe cases in our cohort occurred during the early phase of the pandemic, when clinical experience and evidence-based management strategies were still evolving [24].

All COVID–19-related symptoms—including fever, cough, sore throat, anosmia, dyspnea, and myalgia—were significantly more frequent among hospitalized patients. Bilateral pneumonia was diagnosed exclusively in this group, affecting 25% of those admitted. A history of preexisting respiratory disease was also identified as a risk factor for hospitalization. As expected, patients requiring admission had more severe clinical presentations and symptomatologies.

### 4.4. Obstetric Outcomes: Admitted vs. Non-Admitted Pregnant Women

In our study, 75% of hospitalized pregnant women had a gestational age of less than 35 weeks at the time of admission. In contrast to our findings, previous studies, such as those by Lassi et al. [3] and Donati et al. [14], have reported higher infection and hospitalization rates during the second and third trimesters.

Our data did not demonstrate a significantly higher frequency of obstetric complications among hospitalized pregnant women with COVID-19 compared to those who were not admitted. The preeclampsia rate in hospitalized patients was 12.5%, higher than the 8.8% observed in the non-hospitalized group, although this difference did not reach statistical significance. Several studies have established an association between SARS-CoV-2 infection and an increased risk of preeclampsia, particularly in severe cases, as shown in meta-analyses by Wei et al. [9] and Conde-Agudelo et al. [25]. The limited number of preeclampsia cases in our cohort may have reduced the ability to detect significant differences.

We found that pregnant women with COVID-19 who required hospitalization had a significantly higher rate of cesarean delivery and labor induction. These findings may indicate that deliveries were expedited due to maternal complications associated with infection. While most studies have not identified a consistent association between COVID-19 infection and mode of delivery [6,14,20], some evidence suggests that cesarean birth is more common in cases of moderate to higher disease severity [18], which is consistent with our results.

We also observed a higher incidence of preterm birth among hospitalized pregnant women (15.6% vs. 8.8%), although this difference did not reach statistical significance, likely due to the relatively small number of hospitalized patients. Nonetheless, the existing literature consistently reports increased odds of preterm birth in pregnancies complicated by SARS-CoV-2 infection, particularly in cases involving moderate-to-severe illness [9,20,26]. A national study conducted in Slovakia analyzed SARS-CoV-2 positive cases throughout the entire pandemic period (March 2020–May 2023), also reporting an increased incidence of cesarean deliveries and preterm births among pregnant women infected [8].

As expected, the need for ICU admission was greater among hospitalized patients, further reflecting the more severe clinical course of the infection. The ICU admission rate in our cohort was 9.7%, slightly lower than the 12.8% reported by Metz et al. [20].

### 4.5. Neonatal Outcomes in Pregnant Women with COVID-19: Admitted vs. Non-Admitted Pregnancies

Meta-analyses by Allotey et al. [5] and Smith et al. [12] have reported a significant increase in neonatal mortality among infants born to mothers with COVID-19. Specifically, Allotey et al. [5] documented a neonatal mortality rate of 0.5%. In our study, no neonatal deaths were recorded during the study period. However, neonates born to women hospitalized with COVID-19 exhibited a higher incidence of adverse outcomes, including lower 1 min Apgar scores, increased rates of NICU admission, and a greater frequency of neonatal complications. These findings are consistent with previous studies that have also documented elevated NICU admission rates among neonates born to women with SARS-CoV-2 infection, particularly in cases of severe maternal illness [5,6,20,26]. A meta-analysis by Deng et al. [27] further supports this, showing that neonates born during periods dominated by more transmissible variants—such as Delta and Omicron—had significantly higher risks of adverse outcomes, including NICU admission and respiratory complications, compared to those born during the early (wild-type) phase of the pandemic.

In contrast to the findings of Berumen-Lechuga et al. [26], who reported significantly lower neonatal birth weights in pregnancies complicated by severe COVID-19, our study did not identify any statistically significant differences in birth weight between neonates born to hospitalized and non-hospitalized mothers.

Our findings are consistent with reports from other European countries, where the pandemic has been shown to negatively affect not only obstetric and neonatal outcomes but also the psychological well-being of pregnant individuals (e.g., increased anxiety, stress, and depressive symptoms) [28,29,30]. These studies support the need for comprehensive perinatal care that addresses both medical and psychological aspects during pandemics.

### 4.6. Severity of COVID-19 in Hospitalized Pregnant Women

The rate of severe COVID-19 in our study—defined as ICU admission, mechanical ventilation, and/or maternal death—was 16.1%, slightly higher than the rates reported by Boettcher et al. [6] (5.4–12%) and Schell et al. [31] (10%). Other authors, such as Jering et al. [23], have described lower rates (3.3%) among women admitted for delivery.

In our cohort, obesity (BMI ≥ 30) and non-Spanish nationality were significantly associated with severe cases of COVID-19. Obesity has also been identified as a risk factor for severe disease in pregnant populations, as documented in multiple studies [5,12,32]. Our findings also align with those of Donati et al. [14] and Engjom et al. [32], who identified that foreign nationality—particularly among migrant women—was associated with an increased risk of adverse maternal outcomes. Nevertheless, it must be taken into account that, while nationality was associated with an increased risk of severe maternal COVID-19 in our study, it likely serves as a proxy for unmeasured structural and social factors, such as socioeconomic disadvantage, migrant status, language or cultural barriers, and access to healthcare. It should not be interpreted as a biological or ethnic determinant, and future research should incorporate more nuanced indicators of social and ethnic vulnerability to better understand these disparities.

Additionally, a study conducted in the United States in 2023 [6] identified several pregestational comorbidities, including diabetes, hypertension, and cardiovascular disease, as significant risk factors for maternal mortality, which were included within the definition of severe COVID-19 in our study. However, in our cohort, these comorbidities did not reach statistical significance, possibly due to the limited number of severe cases in our study.

### 4.7. Vaccination

Vaccination against SARS-CoV-2 was identified as a protective factor against COVID-19-related hospitalization in our cohort. A significantly higher proportion of hospitalized pregnant women were unvaccinated. This finding is consistent with previous studies that have recognized unvaccinated status as a significant risk factor for the development of severe COVID-19 during pregnancy [26,32].

In our study, 62.2% of pregnant women had received at least one dose of a COVID-19 vaccine. By the end of the second wave of the pandemic, vaccination coverage among pregnant women varied widely across Europe, ranging from 20% in Lombardy, Italy, to 80% in Norway [32,33]. These differences in vaccination uptake highlight the need for consistent international guidance. In its 2024 interim guidance, the World Health Organization reaffirmed that COVID-19 vaccination during pregnancy is both safe and strongly recommended, particularly for women at high risk of severe disease [34].

These findings reinforce the importance of promoting COVID-19 vaccination among pregnant women, particularly in light of ongoing viral mutations and potential future waves. Recent evidence also supports the safety and effectiveness of booster doses during pregnancy. In a recent multicenter study, Barros et al. [35] reported that newborns of booster-vaccinated mothers had a significantly lower risk of SARS-CoV-2 infection, as well as reduced rates of preterm birth, neonatal respiratory distress, and NICU stay. 

Lastly, it is important to note that although our results suggest a protective effect of COVID-19 vaccination during pregnancy, detailed data regarding the number of doses received, type of vaccine administered, and gestational timing were not consistently available due to limitations in retrospective documentation. This limits the depth of our analysis and should be considered when interpreting the findings.

### 4.8. Strengths and Limitations

A major strength of this study is its focus on the epidemiological and clinical impact of COVID-19 in pregnant women in Spain, a country that experienced one of the highest burdens of infection, hospitalization, and mortality during the pandemic. While extensive data exist regarding the general population, obstetric outcomes related to COVID-19 in Spain have remained underexplored. This study helps fill that gap by providing valuable longitudinal data on maternal and neonatal outcomes across multiple phases of the pandemic, contributing to a more comprehensive understanding of the obstetric implications of SARS-CoV-2 infection in a high-risk setting.

However, several limitations must be acknowledged. First, this was a single-center (monocentric) study conducted at a tertiary-care hospital, which may limit the generalizability of the findings to other settings or healthcare systems. Second, due to its retrospective nature, data collection relied on the accuracy and completeness of medical records, which may have resulted in missing, inconsistent, or erroneous information. Third, selection bias is possible, as case identification depended on available documentation and coding, which may have led to missed or misclassified cases. For all the reasons mentioned above, and although we acknowledge the importance of adjusted models for controlling potential confusion, multivariable logistic regression was not performed due to limitations in data completeness and the small number of events for certain outcomes, which could have compromised the reliability of the estimates. This represents an inherent limitation of our retrospective design and should be taken into account when interpreting the findings.

Lastly, given that the study was conducted at a single tertiary referral center specializing in high-risk pregnancies, the study population may disproportionately represent moderate-to-severe cases of COVID-19. As a result, mild or asymptomatic cases—particularly those managed in outpatient settings—may be underrepresented, potentially limiting the generalizability of the findings.

Another important point to consider is that some comparisons—particularly those involving subgroups such as hospitalized patients—may have lacked sufficient statistical power to detect significant differences due to limited sample sizes. This limitation should be taken into account when interpreting nonsignificant results, as a type II error cannot be ruled out. This aspect has been addressed throughout the Section 4. Moreover, it is important to acknowledge that this study spans a four-year period, during which significant changes were introduced in the clinical management of COVID-19, including the availability of vaccination and updates in treatment protocols. These evolving healthcare strategies may have influenced the observed obstetric and neonatal outcomes across different phases of the pandemic. This limitation should be taken into account when interpreting the results, as the heterogeneity in clinical practices over time may have introduced confounding factors.

Additional limitations include the lack of genomic confirmation for SARS-CoV-2 variant classification, which was based on prevailing epidemiological phases, given that routine genomic sequencing was not available for individual cases during the study period; the absence of long-term follow-up data on neonatal outcomes; and the potential for case misclassification due to reliance on retrospective clinical coding. Moreover, maternal mental health data were not systematically collected, limiting our ability to evaluate the psychological dimension of COVID-19 during pregnancy, which has been associated with adverse outcomes, such as preterm birth, low birth weight, and increased NICU admission. These limitations should be considered when interpreting the findings and their generalizability.

Further multicenter studies with broader geographic representation and prospective designs are warranted to confirm these findings and guide global obstetric management in future pandemics or SARS-CoV-2 resurgence scenarios.

## 5. Conclusions

This study demonstrates that hospital admission due to COVID-19 during pregnancy is associated with an increased risk of adverse obstetric and neonatal outcomes. Specifically, higher rates of preterm birth, labor induction, and cesarean delivery were observed among hospitalized women. Moreover, neonates born to these patients experienced more complications, including lower 1 min Apgar scores and increased NICU admission rates. These findings underscore the need for vigilant monitoring, early intervention, and the promotion of COVID-19 vaccination during pregnancy to mitigate risks for both mothers and newborns.

## Figures and Tables

**Table 1:** (**a**) Gestational age at delivery in pregnant women with and without COVID-19, stratified by gestational age and study period. (**b**) Obstetric outcomes across different phases of the study period.

**(a)**						
	**Gestational Age at Birth** **<28 Weeks** **28–37 Weeks** **≥37 Weeks**					
**Period of Study**	**Overall** **% (n/N) ^a^**	**COVID-19** **% (n/N^1^)**		**Non-COVID-19** **% (n/N^2^)**		***p*-Value**
4 March 2020–28 February 2021	1.11 (21/1894) 8.08 (153/1894) 90.81 (1720/1894)	5.56 (2/36) 36.11 (13/36) 58.33 (21/36)		1.02 (19/1858) 7.53 (140/1858) 91.44 (1699/1858)		**<0.001**
1 March 2021–31 December 2021	0.74 (12/1630) 8.83 (144/1630) 90.43 (1474/1630)	0 (0/18) 22.22 (4/18) 77.78 (14/18)		0.74 (12/1612) 8.68 (140/1612) 90.57 (1460/1612)		0.125
1 January 2022–5 May 2023	0.96 (25/2596) 7.82 (203/2596) 91.22 (2368/2596)	0 (0/186) 13.44 (25/186) 86.56 (161/186)		1.04 (25/2410) 7.39 (178/2410) 91.58 (2207/2410)		**0.005**
Overall	0.95 (58/6120) 8.17 (500/6120) 90.88 (5562/6120)	0.83 (2/240) 17.5 (42/240) 81.67 (196/240)		0.95 (56/5880) 7.79 (458/5880) 91.26 (5366/5880)		**<0.001**
**(b)**						
	**Cesarean Section**			**Induced Labor**		
**Period of study**	**COVID-19** **% (n/N^1^)**	**No COVID-19** **% (n/N^2^)**	***p*-value**	**COVID-19** **% (n/N^1^)**	**No COVID-19** **% (n/N^2^)**	***p*-value**
4 March 2020–28 February 2021	36.1 (13/36)	23.3 (433/1858)	0.072	47.2 (17/36)	40.3 (749/1858)	0.441
1 March 2021–31 December 2021	33.3 (6/18)	23.8 (384/1612)	0.181	66.7 (12/18)	41.1 (662/1612)	**0.028**
1 January 2022–5 May 2023	27.4 (51/186)	24.1 (582/2410)	0.321	41.4 (77/186)	34.9 (842/2410)	0.075
Overall	29.2 (70/240)	23.8 (1399/5880)	0.052	44.2 (106/240)	38.3 (2253/5880)	0.521

^a^ Data are presented as percentages (%), number of events (n), and total number of cases (N). N^1^: total number of deliveries in women with COVID-19; N^2^: total number of deliveries in women without COVID-19. In bold: *p* < 0.05.

**Table 2:** Epidemiological characteristics, comorbidities, clinical symptoms, complications, and outcomes in pregnant women hospitalized with COVID-19.

	Overall (n = 249) % (n/N) ^a^	Non-Admitted (n = 217) % (n/N) ^a^	Admitted (n = 32) % (n/N) ^a^	*p*-Value
**Age**. Mdn (IQR)	32 (26–36)	31 (26–36)	33 (27.5–36.5)	0.251
**Country of birth**				
Spain	62.7 (156/249)	64.5 (140/217)	50 (16/32)	0.113
South America	21.7 (54/249)	20.7 (45/217)	28.1 (9/32)	
Africa	12.4 (31/249)	11.1 (24/217)	21.9 (7/32)	
Europe	2.8 (7/249)	3.2 (7/217)	0 (0/32)	
**Period of study**				**<0.001**
4 March 2020–28 February 2021	15.7 (39/249)	12.4 (27/217)	37.5 (12/32)	
1 March 2021–31 December 2021	8.0 (20/249)	6.9 (15/217)	15.6 (5/32)	
1 January 2022–5 May 2023	76.3 (190/249)	80.6 (175/217)	46.9 (15/32)	
**Comorbidities**				
Obesity *	20.6 (50/243)	20.9 (44/211)	19.4 (6/31)	0.847
Pulmonary disease	6.4 (16/249)	5.1 (11/217)	15.6 (5/32)	**0.040**
Other comorbidities **	26.9 (67/249)	25.8 (56/217)	34.4 (11/32)	0.308
**Clinical symptoms**				
Fever	40.5 (94/232)	36.5 (73/200)	65.6 (21/32)	**<0.001**
Cough	32 (74/231)	27 (54/200)	64.5 (20/31)	**<0.001**
Myalgia	19.8 (46/232	17.5 (35/200)	34.4 (11/32)	**0.026**
Odynophagia	18.1 (42/232)	14.5 (29/200)	40.6 (13/32)	**<0.001**
Dyspnea	12 (28/233)	4.5 (9/201)	59.4 (19/32)	**<0.001**
Anosmia	3.4 (8/233)	2 (4/201)	12.5 (4/32)	**0.014**
**Clinical complications**				
Bilateral pneumonia	10.0 (25/249)	0 (0/217)	25 (8/32)	**<0.001**
Thromboembolism	0.4 (1/249)	0.5 (1/217)	0 (0/32)	1.000
**Outcomes**				
Admission ICU	1.2 (3/248)	0 (0/217)	9.7 (3/31)	**0.002**
NIMV	0.8 (2/248)	0 (0/217)	6.4 (2/31)	**0.015**
Death	0.4 (1/248)	0 (0/217)	3.2 (1/31)	0.120

^a^ Data are presented as percentages (%), number of events (n), and total number of events (N). Continuous variables are shown as median (Mdn) and interquartile range (IQR) * BMI ≥ 30. ** Other comorbidities: chronic hypertension, pregestational diabetes, or history of previous spontaneous abortion. Abbreviations: Mdn, median; IQR, interquartile range; NIMV, non-invasive mechanical ventilation; ICU, intensive care unit. In bold: *p* < 0.05.

**Table 3:** Comparative analysis of obstetric outcomes according to hospital admission.

	Overall (n = 249) % (n/N) ^a^	Non-Admitted (n = 217) % (n/N) ^a^	Admitted (n = 32) % (n/N) ^a^	*p*-Value
**Gestational age** ** at the time of infection**				
Mdn (IQR)	32 (22–38)	32 (21–38)	29.50 (23.75–34.5)	0.160
<24 weeks	27.6 (68/246)	28 (60/214)	25 (8/32)	**0.037**
24–34 weeks	30.9 (76/246)	28 (60/214)	50 (16/32)	
≥35 weeks	41.5 (102/246)	43.9 (94/214)	25 (8/32)	
**Obstetric history**				
Number of pregnancies, Mdn (IQR)	2 (1–3)	2 (1–3)	2 (1–3.75)	0.657
Number of previous miscarriages	30.1 (75/249)	30.9 (67/217)	25 (8/32)	0.499
Previous vaginal deliveries	38.2 (95/249)	38.7 (84/217)	34.4 (11/32)	0.637
Previous cesarean sections	15.8 (39/247)	15.3 (33/216)	19.4 (6/31)	0.598
**Pregnancy complications**				
Gestational diabetes	11.2 (28/249)	11.5 (25/217)	9.4 (3/32)	1.000
Hypertensive disorders	9.2 (23/249)	8.8 (19/217)	12.5 (4/32)	0.520
Threatened of preterm labor	5.4 (13/217)	5.7 (12/210)	3.2 (1/31)	1.000
Fetal pathology *	5 (12/242)	4.8 (10/210)	6.3 (2/32)	0.663
**Delivery outcomes**				
Gestational age at birth, weeks. Mdn. (IQR)	39 (26–38)	39 (37–40)	38 (37–39)	0.251
<34 weeks	4.6 (11/240)	3.8 (8/208)	9.4 (3/32)	0.121
≥34 weeks	95.4 (229/240)	96.2 (200/208)	90.6 (29/32)	0.168
Term delivery	86.7 (216/249)	87.1 (189/217)	84.4 (27/32)	0.437
Induced labor	42.2 (105/249)	39.6 (86/217)	59.4 (19/32)	**0.023**
Unsatisfactory intrapartum fetal cardiotocography	11.3 (26/230)	10.6 (21/199)	16.1 (5/31)	0.363
Cesarean section	27.7 (69/249)	24.9 (54/217)	46.9 (15/32)	**0.009**
Postpartum hemorrhage	4.9 (11/224)	5.1 (10/195)	3.4 (1/29)	1.000
**Maternal complications**				
Maternal blood transfusion	0.4 (1/239)	0.5 (1/207)	0 (0/32)	1.000
Maternal admission to ICU	1.3 (3/238)	0 (0/207)	9.7 (3/31)	**0.002**
Postpartum fever	2.5 (6/239)	2.9 (6/207)	0 (0/32)	1.000

^a^ Data are presented as percentages (%), number of events (n), and total number of events (N). Continuous variables are shown as median (Mdn) and interquartile range (IQR). * Fetal pathology: includes congenital malformations and/or fetal growth abnormalities. Abbreviations: Mdn: median; IQR: interquartile range; ICU: intensive care unit. In bold: *p* < 0.05.

**Table 4:** Characteristics of newborns of pregnant women with COVID-19 according to hospital admission.

	Overall % (n/N) ^a^	Non-Admitted % (n/N) ^a^	Admitted % (n/N) ^a^	*p*-Value
Birth weight; g				
Mdn (IQR)	3168 (2920–3501)	3248 (2937–3542)	3.090 (2837–3535)	0.132
<2500 g	6.3 (15/223)	5.8 (12/194)	9.4 (3/29)	0.433
Apgar 1 score (1st min)				**<0.001**
<7	5.0 (12/240)	2.9 (6/208)	18.7 (6/32)	
≥7	95.0 (228/240)	97.1 (202/208)	81.3 (26/32)	
Apgar score (5th min)				**0.133**
<7	0.4 (1/240)	0	3.1 (1/32)	
≥7	99.6 (239/240)	100 (208/208)	96.9 (31/32)	
Newborn admitted in NICU	10.5 (25/238)	8.2 (17/207)	25.8 (8/31)	**0.007**
Neonatal complications *	9.7 (23/237)	7.7 (16/207)	23.3 (7/30)	**0.015**
Umbilical arterial pH	7.25 (7.24–7.27)	7.25 (7.24–7.28)	7.25 (7.23–7.29)	0.921
Umbilical venous pH	7.33 (7.27–7.37)	7.33 (7.28–7.36)	7.32 (7.28–7.34)	0.585

^a^ Data are presented as percentages (%), number of events (n), and total number of events (N). Continuous variables are shown as median (Mdn) and interquartile range (IQR). * Neonatal complications include respiratory distress syndrome, necrotizing enterocolitis, neonatal sepsis, and hypoxic–ischemic encephalopathy. Abbreviations: Mdn: median; IQR: interquartile range; NICU: neonatal intensive care unit. In bold: *p* < 0.05.

**Table 5:** Risk factors for severe COVID-19 in hospitalized pregnant women.

	Severe COVID-19 % (n/N) ^a^	Non-Severe COVID-19 % (n/N) ^a^	Crude OR	*p*-Value
Age ≥ 35 years	0 (0/5)	38.5 (10/26)	NA	0.147
Gestational age at delivery, (weeks)				1.000
<35	80.0 (4/5)	74.1 (20/27)	1	
≥35	20.0 (1/5)	27.9 (7/26)	0.71 (0.07–7.6)	
Born in Spain	0 (0/5)	61.5 (16/26)	NA	**0.018**
**Comorbidities**				
Obesity *	60.0 (3/5)	12.0 (3/25)	11.0 (1.27–95)	**0.041**
Pulmonary diseases	20.0 (1/5)	11.5 (3/26)	1.91 (0.15–23)	0.525
Hypertension	0	15.4 (4/26)	NA	1.000
**Clinical presentation**				
Fever	100 (5/5)	61.5 (16/26)	NA	0.147
Cough	75.0 (3/4)	65.4 (17/26)	1.58 (0.14–17)	1.000
Odynophagia	6.0 (3/5)	38.5 (10/26)	2.40 (0.33–17)	0.625
Anosmia	0 (0/5)	15.4 (4/26)	NA	1.000
Dyspnea	100 (5/5)	53.8 (14/26)	NA	0.128
Myalgia	60 (3/5)	30.8 (8/26)	3.37 (0.46–24)	0.317
**Treatment**				
LMWH	80 (4/5)	84.6 (22/26)	0.77 (0.64–8.31)	1.000
Steroid	100 (5/5)	26.9 (7/26)	NA	**0.005**
**Complication/outcome**				
Bilateral pneumonia	100 (5/5)	11.5 (3/26)	NA	**<0.001**
Thromboembolism	0	3.8 (1/26)	NA	1.000
Death	20 (1/5)	0	NA	0.161

^a^ Data are presented as percentages (%), number of events (n), and total number of events (N). Continuous variables are expressed as median (Mdn) and interquartile range (IQR). * BMI ≥ 30. Abbreviations: LMWH, low-molecular-weight heparin. In bold: *p* < 0.05.

**Table 6:** Comparative analysis of hospital admissions according to vaccination status.

	Overall % (n/N) ^a^	Non-Admitted % (n/N) ^a^	Admitted % (n/N) ^a^	*p*-Value
**Vaccination status**				
Not vaccinated	47.4 (118/249)	43.8 (95/217)	71.9 (23/32)	**0.003**
Vaccinated (1 or 2 doses)	52.6 (131/249)	56.2 (122/217)	28.1 (9/32)	

^a^ Data are presented as percentages (%), number of events (n), and total number (N). In bold: *p* < 0.05.

## Data Availability

The original contributions presented in this study are included in the article. Further inquiries can be directed to the corresponding author.

## Abbreviations

The following abbreviations are used in this manuscript:

BMI	Body mass index
CI	Confidence interval
DBGUH	Doctor Balmis General University Hospital
ICU	Intensive care unit
IMV	Invasive mechanical ventilation
IQR	Interquartile range
IUGR	Intrauterine growth restriction
LMWH	Low-molecular-weight heparin
Mdn	Median
NICU	Neonatal intensive care unit
NIMV	Non-invasive mechanical ventilation
OR	Odds ratio
RT-PCR	Reverse transcription polymerase chain reaction
SD	Standard deviation
SGA	Small for gestational age
WHO	World Health Organization

## References

[1] Ellington S., Strid P., Tong V.T., Woodworth K., Galang R.R., Zambrano L.D., Nahabedian J., Anderson K., Gilboa S.M. (2020). Characteristics of Women of Reproductive Age with Laboratory-Confirmed SARS-CoV-2 Infection by Pregnancy Status—United States, January 22–June 7, 2020. MMWR Morb. Mortal. Wkly. Rep..

[2] Royal College of Obstetricians and Gynaecologists (2022). Coronavirus (COVID-19) Infection in Pregnancy. Information for healthcare professionals.

[3] Lassi Z.S., Ana A., Das J.K., Salam R.A., Padhani Z.A., Irfan O., Bhutta Z.A. (2021). A systematic review and meta-analysis of data on pregnant women with confirmed COVID-19: Clinical presentation, and pregnancy and perinatal outcomes based on COVID-19 severity. J. Glob. Health..

[4] Molina R.L., Tsai T.C., Dai D., Soto M., Rosenthal N., Orav E.J., Figueroa J.F. (2022). Comparison of Pregnancy and Birth Outcomes Before vs During the COVID-19 Pandemic. JAMA Netw. Open..

[5] Allotey J., Stallings E., Bonet M., Yap M., Chatterjee S., Kew T., Debenham L., Llavall A.C., Dixit A., Zhou D. (2020). Clinical manifestations, risk factors, and maternal and perinatal outcomes of coronavirus disease 2019 in pregnancy: Living systematic review and meta-analysis. BMJ.

[6] Boettcher L.B., Metz T.D. (2023). Maternal and neonatal outcomes following SARS-CoV-2 infection. Semin. Fetal Neonatal Med..

[7] Pineles B.L., Goodman K.E., Pineles L., O’HAra L.M., Nadimpalli G., Magder L.S., Baghdadi J.D., Parchem J.G., Harris A.D. (2022). Pregnancy and the Risk of In-Hospital Coronavirus Disease 2019 (COVID-19) Mortality. Obstet. Gynecol..

[8] Goldbergerová A., Kováč L., Marcišová C., Borovský M., Kotríková D., Izáková Ľ., Mikas J., Námešná J., Krištúfková Z., Krištúfková A. (2024). Assessing the Impact of COVID-19 on Pregnancy and Maternal Outcomes: A Slovak National Study. Reprod. Med..

[9] Wei S.Q., Bilodeau-Bertrand M., Liu S., Auger N. (2021). The impact of COVID-19 on pregnancy outcomes: A systematic review and meta-analysis. Can. Med Assoc. J..

[10] Mullins E., Perry A., Banerjee J., Townson J., Grozeva D., Milton R., Kirby N., Playle R., Bourne T., Lees C. (2022). Pregnancy and neonatal outcomes of COVID-19: The PAN_COVID study. Eur. J. Obstet. Gynecol. Reprod. Biol..

[11] Incognito G., Distefano R.E., Campo G., Gulino F.A., Gulisano C., Gullotta C., Gullo G., Cucinella G., Tuscano A., Bruno M.T. (2023). Comparison of Maternal and Neonatal Outcomes between SARS-CoV-2 Variants: A Retrospective, Monocentric Study. J. Clin. Med..

[12] Smith E.R., Oakley E., Grandner G.W., Rukundo G., Farooq F., Ferguson K., Baumann S., Waldorf K.M.A., Afshar Y., Ahlberg M. (2023). Clinical risk factors of adverse outcomes among women with COVID-19 in the pregnancy and postpartum period: A sequential, prospective meta-analysis. Am. J. Obstet. Gynecol..

[13] Son M., Gallagher K., Lo J.Y., Lindgren E.J., Burris H.H., Dysart K., Greenspan J., Culhane J.F., Handley S.C.M. (2021). Coronavirus Disease 2019 (COVID-19) pandemic and pregnancy outcomes in a U. S. population. Obstet. Gynecol..

[14] Donati S., Corsi E., Maraschini A., Salvatore M.A., the ItOSS-COVID-19 Working Group (2022). SARS-CoV-2 infection among hospitalised pregnant women and impact of different viral strains on COVID-19 severity in Italy: a national prospective population-based cohort study. BJOG.

[15] Vousden N., Ramakrishnan R., Bunch K., Morris E., Simpson N., Gale C., O’Brien P., Quigley M., Brocklehurst P., Kurinczuk J.J. (2023). Impact of different SARS-CoV-2 variants on maternal and perinatal outcomes: A population-based study in England. BMJ.

[16] Chmielewska B., Barratt I., Townsend R., Kalafat E., van der Meulen J., Gurol-Urganci I., O’Brien P., Morris E., Draycott T., Thangaratinam S. (2021). Effects of the COVID-19 pandemic on maternal and perinatal outcomes: A systematic review and meta-analysis. Lancet Glob. Health..

[17] Seaton C.L., Cohen A., Henninger E.M., Gendlina I., Hou W., Bernstein P.S., Duong T.Q. (2023). Coronavirus disease 2019 (COVID-19) perinatal outcomes across the pandemic at an academic medical center in New York City. Obstet. Gynecol..

[18] Hui L., Marzan M.B., Rolnik D.L., Potenza S., Pritchard N., Said J.M., Palmer K.R., Whitehead C.L., Sheehan P.M., Ford J. (2023). Reductions on stillbirths and preterm birth in COVID-19 vaccinated women: A multicenter cohort study of vaccination uptake and perinatal outcomes. Am. J. Obstet. Gynecol..

[19] Parazzini F., Bartolus R., Mauri P.A., Favilli A., Gerli S., Ferrazzi E. (2020). Delivery in pregnant women infected with SARS-CoV-2: A fast review. Int. J. Gynaecol. Obstet..

[20] Metz T.D., Clifton R.G., Hughes B.L., Sandoval G.J., Grobman W.A., Saade G.R., Manuck T.A., Longo M., Sowles A., Clark K. (2022). Association of SARS-Cov-2 infection with serious maternal morbidity and mortality from obstetric complications. JAMA.

[21] Morán Antolín E., Broullón Molanes J.R., de la Cruz Conty M.L., Pardilla M.B.E., Martín M.d.P.G., Bueno J.A.S., Acebal L.F., Recarte P.P., Bartolomé A.Á., Cendán J.P.M. (2021). SARS-CoV-2 infection and C-section: A prospective observational study. Virus.

[22] Trinh L.T.T., Achat H.M., Pesce A. (2023). Caesarean sections before and during the COVID-19 pandemic in western Sydney, Australia. J. Obstet. Gynaecol..

[23] Jering K.S., Claggett B.L., Cunningham J.W., Rosenthal N., Vardeny O., Greene M.F., Solomon S.D. (2021). Clinical characteristics and outcomes of hospitalized women giving birth with and without COVID-19. JAMA Intern. Med..

[24] Pinargote-Celorio H., Moreno-Pérez Ó., González-De-La-Aleja P., Llenas-García J., Pérez-Crespo P.M.M., Rodríguez-Díaz J.-C., Martínez-López B., Gutiérrez N.M., Ramos-Rincón J.-M., Merino E. (2024). Real-world effectiveness of early anti-SARS therapy in severely immunocompromised COVID-19 outpatients during the SARS-CoV-2 omicron variant era: A propensity score-adjusted retrospective cohort study. J. Antimicrob. Chemother..

[25] Conde-Agudelo A., Romero R. (2022). SARS-CoV-2 infection during pregnancy and risk of preeclampsia: A systematic review and meta-analysis. Am. J. Obstet. Gynecol..

[26] Berumen-Lechuga M.G., Leaños-Miranda A., Molina-Pérez C.J., García-Cortés L.R., Palomo-Piñón S. (2023). Risk factors for severe-critical COVID-19 in pregnant women. J. Clin. Med..

[27] Deng J., Ma Y., Liu Q., Du M., Liu M., Liu J. (2022). Association of infection with different SARS-CoV-2 variants during pregnancy with maternal and perinatal outcomes: A systematic review and meta-analysis. Int. J. Environ. Res. Public Health.

[28] La Rosa V.L., Oddo-Sommerfeld S., Schermelleh-Engel K., Commodari E. (2024). From lockdown to cradle: Navigating the psychological challenges of childbirth during the COVID-19 pandemic in Italy–Evidence from a 3-year analysis. Curr. Psychol..

[29] Giesbrecht G.F., Rojas L., Patel S., Kuret V., MacKinnon A.L., Tomfohr-Madsen L., Lebel C. (2022). Fear of COVID-19, mental health, and pregnancy outcomes in the pregnancy during the COVID-19 pandemic study: Fear of COVID-19 and pregnancy outcomes. J. Affect. Disord..

[30] Tauqeer F., Ceulemans M., Gerbier E., Passier A., Oliver A., Foulon V., Panchaud A., Lupattelli A., Nordeng H. (2023). Mental health of pregnant and postpartum women during the third wave of the COVID-19 pandemic: A European cross-sectional study. BMJ Open.

[31] Schell R.C., Macias D.A., Garner W.H., White A.M., McIntire D.D., Pruszynski J., Adhikari E.H. (2022). Examining the impact of trimester of diagnosis on COVID-19 disease progression in pregnancy. Am. J. Obstet. Gynecol. MFM..

[32] Engjom H., Aabakke A.J.M., Klungsoyr K., Svanvik T., Äyräs O., Jonasdottir E., Thurn L., Jones E., Pettersson K., Nyfløt L.T. (2021). COVID-19 in pregnancy-characteristics and outcomes of pregnant women admitted to hospital because of SARS-CoV-2 infection in the Nordic countries. Acta Obstet. Gynecol. Scand..

[33] Carbone L., Trinchillo M.G., Di Girolamo R., Raffone A., Saccone G., Iorio G.G., Gabrielli O., Maruotti G.M. (2022). COVID-19 vaccine and pregnancy outcomes: A systematic review and meta-analysis. Int. J. Gynaecol. Obstet..

[34] World Health Organization (WHO) Interim Recommendations for the Use of COVID-19 Vaccines in Pregnant and Lactating Women—March 2024. https://www.who.int.

[35] Barros F.C., Gunier R.B., Rego A., Sentilhes L., Rauch S., Gandino S., Teji J.S., Thornton J.G., Kachikis A.B., Nieto R. (2024). Maternal vaccination against COVID-19 and neonatal outcomes during Omicron: INTERCOVID-2022 study. Am. J. Obstet. Gynecol..

